# Comprehensive Analysis of Ultrasonic Vocalizations in a Mouse Model of Fragile X Syndrome Reveals Limited, Call Type Specific Deficits

**DOI:** 10.1371/journal.pone.0044816

**Published:** 2012-09-11

**Authors:** Snigdha Roy, Nick Watkins, Detlef Heck

**Affiliations:** 1 Department of Anatomy and Neurobiology, University of Tennessee Health Science Center, Memphis, Tennessee, United States of America; 2 Department of Biology, Christian Brothers University, Memphis, Tennessee, United States of America; Wake Forest University, United States of America

## Abstract

Fragile X syndrome (FXS) is a well-recognized form of inherited mental retardation, caused by a mutation in the fragile X mental retardation 1 (Fmr1) gene. The gene is located on the long arm of the X chromosome and encodes fragile X mental retardation protein (FMRP). Absence of FMRP in fragile X patients as well as in *Fmr1* knockout (KO) mice results, among other changes, in abnormal dendritic spine formation and altered synaptic plasticity in the neocortex and hippocampus. Clinical features of FXS include cognitive impairment, anxiety, abnormal social interaction, mental retardation, motor coordination and speech articulation deficits. Mouse pups generate ultrasonic vocalizations (USVs) when isolated from their mothers. Whether those social ultrasonic vocalizations are deficient in mouse models of FXS is unknown. Here we compared isolation-induced USVs generated by pups of *Fmr1*-KO mice with those of their wild type (WT) littermates. Though the total number of calls was not significantly different between genotypes, a detailed analysis of 10 different categories of calls revealed that loss of Fmr1 expression in mice causes limited and call-type specific deficits in ultrasonic vocalization: the carrier frequency of flat calls was higher, the percentage of downward calls was lower and that the frequency range of complex calls was wider in *Fmr1*-KO mice compared to their WT littermates.

## Introduction

Fragile X syndrome affects approximately 1/4000 males. Due to the mosaicism of the x-linked mutation, symptoms are typically milder in females but about 1/8000 females have significant features of the syndrome [1]. Most male fragile X patients have moderate to severe delays in the onset of speech and language. Articulation deficits are found in male and female FXS patients, [2], [3]. Males with FXS do not articulate significantly faster than chronological age (CA)-matched males but use significantly shorter utterances and have tendencies to pause less often than CA-matched males [4]. Speech articulation problems impact their social interactions because the generation of socially meaningful spoken language includes the ability to modulate tone, volume and prosody (ups and downs of the voice). Approximately 30% of male FXS patients also have autism, as determined by the standardized criteria of the Autism Diagnostic Observation Scale (ADOS) and the Autism Diagnostic Interview (ADI-R) [5]–[7]. An additional 30% of boys have pervasive developmental disorder, not otherwise specified (PDD-NOS) [5]. Among the remaining FXS patients who do not meet the criteria for an autism spectrum disorder (ASD), the majority have one or more autistic features [5].

Mice begin vocalizing shortly after birth, with a peak in vocalization rates occurring around postnatal day 8 (P8). They continue vocalizing, albeit at reduced rates, throughout adulthood [8]. Here we investigated whether the loss of *Fmr1* expression causes deficits in ultrasonic vocalizations in mouse pups. USV emission is an important parameter for social communication in mouse pups [9]. A reduced level of calling and an unusual calling pattern have been reported in several mouse models of autism spectrum disorders [10]–[13], which could be indicative of communication impairment in mouse pups isolated from their mothers.

Mouse pups produce different shapes/categories of USVs when they are separated from their mother and siblings [14]. Scattoni et al. [14] classified individual pup calls into 10 categories (Complex, Flat, Frequency Steps, Composite, Shorts, Chevron, Downward, Upward, Two-syllable and Harmonic). We used their classification scheme to categorize and compare pup vocalizations of *Fmr1*-KO and WT mice. Our findings revealed specific qualitative and quantitative deficits in the production of isolation induced USVs in pups of a *Fmr1*-KO mice compared to their wild type littermates.

## Materials and Methods

### Animals and animal care

Breeding pairs of B6.129P2-Fmr1tm1Cgr/J mice were purchased from Jackson Laboratory (stock # 003025) and bred by pairing wild type males with heterozygous females in a conventional mouse vivarium at the University of Tennessee Health Science Center using harem breading trios. Pups were kept with the dam until weaning at Postnatal Day 21 (P21). Juveniles were housed by gender in standard plastic cages not exceeding four per cage. All mice used in this study were raised and all experiments were performed in accordance with procedural guidelines approved by the University of Tennessee Health Science Center Animal Care and Use Committee. Principles of laboratory animal care (NIH publication No. 86–23, rev. 1996) were followed. Only male WT and *Fmr1*-KO littermates were used for testing to avoid variability in behavioral performance because of mosaicism of the mutant allele due to X-inactivation in females. All experiments were performed with 8-day-old pups (6 WT, 6 KO). All mice were weighed before behavioral testing began and the measurements were used for statistical comparison of body weights between *Fmr1*-KO and WT mice. Behavioral testing was performed by investigators blinded to genotype. Recordings were performed during afternoon sessions (1–5 pm) held constant across mice. After completion of behavioral tests, small pieces of tails were collected from the animals and processed for genotyping (Transnetyx, Inc, Cordova, TN).

### Ultrasonic vocalization measurement

For USV recording of social calls [15], 8 day old pups (10 pups for each genotype) were separated from their mothers and placed inside a sound attenuating Styrofoam box. The box had an ultrasound microphone in the center of the lid, with the microphone about 10 cm above the floor. The temperature of the room was maintained at 21°C. Vocalizations were recorded over a 3 minute period (250 kHz, 16 bit) using an ultrasound-recording system (Avisoft Bioacoustics, Berlin, Germany, Sofware Version 3.2). Recorded files were transferred to Avisoft SASLab Pro (Version 4.40) for analysis, and a fast Fourier transformation (FFT) was conducted. Spectrograms were generated with an FFT-length of 1024 points and a time window overlap of 75% (100% Frame, Hamming window). The spectrogram was produced at a frequency resolution of 488 Hz and a time resolution of 1 ms.

Waveform patterns recorded in ten pups from each genotype were examined in depth. We classified a total of 1399 calls from WT and 1543 calls from KO animals. Each call was classified as one of 10 distinct categories, based on internal pitch changes, lengths and shapes, using previously published categorizations [14]. One of the authors of this paper (SR or NW) performed the initial classification of calls based on the spectrogram, while blind to the genotype of the mice. To test repeatability of the classification process a third person naïve to the process and not otherwise involved in the study was asked to classify 265 calls. This third person was also blind to the genotype of mice. Overlap of classification between the third person and the authors was 88.4%.

Besides the counts of call types, other parameters analyzed for each test day included 1) the total number of calls 2) the percentage of calls in the different categories, 3) the duration of calls, 4) the average carrier frequency of calls and 5) the frequency modulation depth of calls. We used power spectral density analysis to determine the frequency at peak power of each call. Average values of all parameters were calculated for each mouse and used for the statistical comparison of *Fmr1*-KO and wild-type mice.

### Statistical analysis

A mixed-model Analysis of Variance (ANOVA) with repeated measures was performed to analyze genotype dependent effects on the ultrasonic vocalizations, with the genotype as factor and call categories as the repeated measures [14]. To analyze genotype differences within call categories, an ANOVA was performed for each category and Newman-Keuls post-hoc comparison followed by Bonferroni correction. Nonparametric analysis (Mann Whitney test) was used to analyze body weight differences. For all comparisons, significance was set at p<0.05.

## Results

We classified all USV calls recorded during a 3 minute observation period into 10 types (Complex, Flat, Frequency Steps, Composite, Shorts, Chevron, Downward, Upward, Two-syllable and Harmonic) according to the classification scheme proposed by Scattoni et al. [14]. A total of 1399 calls from WT mice (N = 10) and 1543 calls from *Fmr1* KO mice (N = 10) were recorded and classified. Based on this classification we identified three deficits in USV calls in pups of *Fmr1* KO mice.

### Increased number of downward call emissions

**Figure 1:**
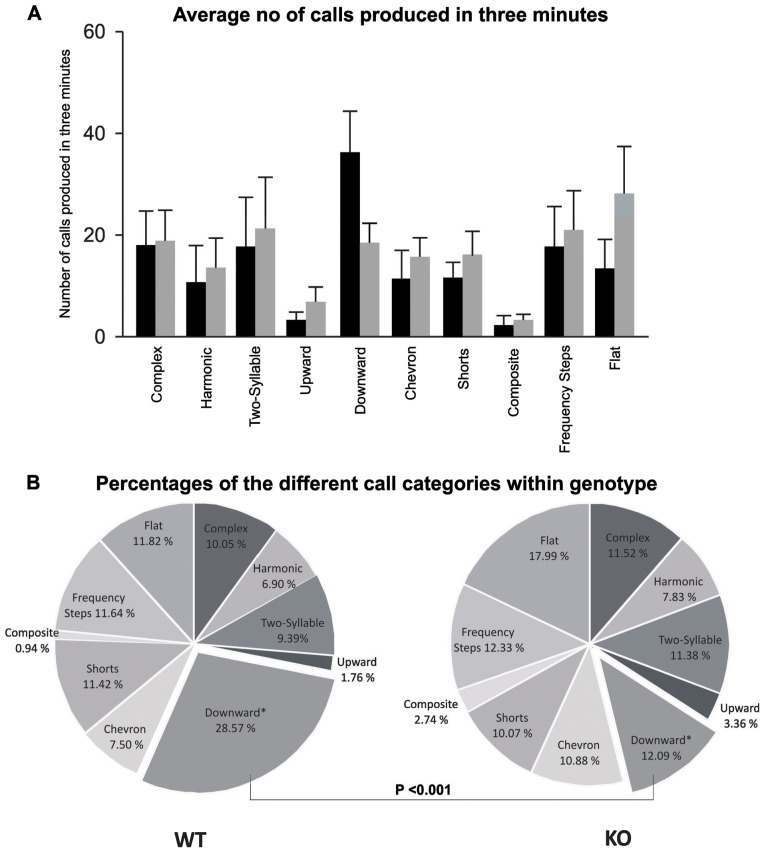
Comparison of different categories of calls produced by WT and *Fmr1*-KO mice: A) Total number and B) The percentages of the different call categories within the genotype. Percentages were calculated in each genotype as: (number of calls in each category/total number of calls analyzed in each subject) * 100. The total number of total calls analyzed across all mice was: WT = 1399; KO = 1543 collected from 10 pups per genotype. The total number of call emissions and the number of calls within a category (including downward calls) were not significantly different between genotypes (A). However, analysis of the percentage of different call categorys revealed that the percentage of downward calls produced by the *Fmr1*-KO pups were significantly lower (p<0.001) compared to their WT littermates (B).

The total number of downward call emissions was reduced in pups of *Fmr1*-KO compared to their WT littermates (Figure 1A), but that difference was not statistically significant. However, analysis of the percentage of downward calls revealed that they accounted for almost 30% of all calls in WT pups but less than 12% in *Fmr1*-KO pups [genotype x %call interaction: F(1, 18) = 3.177, p = 0.001; Newman-Keuls at % downward calls : p<0.001 for WT versus KO; Figure 1B].

### Carrier frequency of flat calls

**Figure 2:**
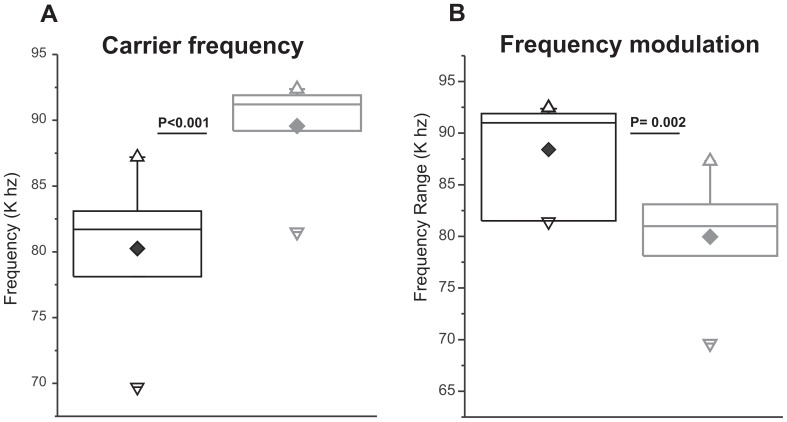
Carrier frequency and frequency modulation of vocalizations during the three minutes of isolation period from mother and littermates: A) Box plot showing the mean carrier frequency of flat calls (Diamonds) together with the 25^th^ to 75^th^ percentile (box height) and median (horizontal line). Triangles at the top and bottom indicate 99 and 1 percentile, respectively. Error bars represent standard error of mean (SE). Mean carrier frequency was calculated from first ten flat calls during isolation. Black outlines: WT littermate control mice; gray outlines: *Fmr1*-KO. B) Box plot as in A) but showing the mean range of frequency modulation of complex calls. Black outlines: WT littermate control mice; gray outlines: *Fmr1*-KO. Dashes (-) mark minimum and maximum values. The average carrier frequency of flat calls emitted by KO mice was higher (p<0.001) than in WT animals (Figure 2A). Call frequency modulation was significantly increased in *Fmr1*-KO mice (p = 0.004) compared to WT pups (Figure 2B).

To compare the average carrier frequencies at which calls were emitted, we compared flat calls, as this category of calls maintains a single carrier frequency throughout the call. The average career frequency measurements were obtained from 10 mice of each genotype, based on averages from 10 flat calls per mouse. The average carrier frequency of flat calls emitted by KO mice (N = 10 mice; mean frequency 88.058 kHz) was higher than in WT animals (N = 10 mice; mean frequency 79.745 kHz) [F (1, 18) = 17.098, p<0.001] (Figure 2A).

### Frequency modulation of complex calls

In order to analyze the frequency modulation of calls, i.e. the width of the frequency band covered by frequency modulation within calls, we measured the difference between the lowest and the highest frequency within calls [9], [11]. For this purpose complex, upward, downward and chevron calls were analyzed as these types of calls show prominent frequency modulation. The average frequency modulation measurements were obtained from 10 calls of each of the 4 call types per mouse. Call frequency modulation was significantly increased in all of the above types of calls in *Fmr1*-KO (N = 10) compared to WT (N = 10) pups [genotype x call interaction: F (1,18) = 5.211, p = 0.004; Newman-Keuls at complex call p = 0.002 for WT versus KO; Figure 2B].

### Duration of calls

**Figure 3:**
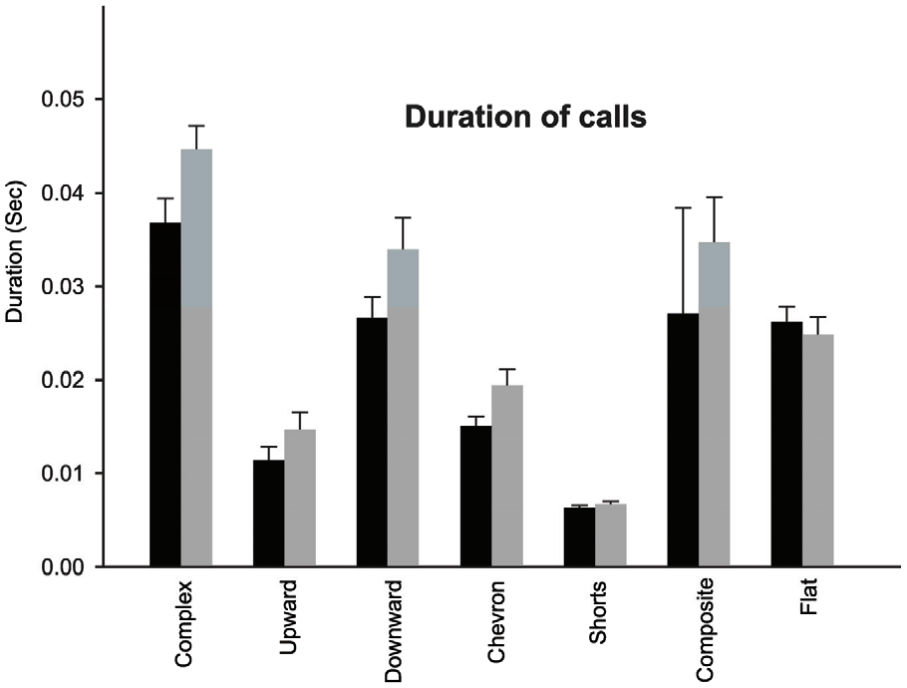
Duration of separation induced ultrasonic vocalizations. The average duration measurements were obtained from 10 mice of each genotype, averaging 10 calls of each type per mouse. Black bar: WT littermates; gray bar: *Fmr1*-KO mice. Error bars represent standard error of mean (SE). There was no significant difference in the duration of any call type between the genotypes.

Duration of calls was measured for complex, upward, downward, chevron, shorts, composite and flat calls (Figure 3). The average duration measurements were obtained from 10 mice of each genotype, averaging 10 calls of each category per mouse. There was no effect of genotypes on the duration of calls for any of those call subtypes (Table 1).

**Table 1:** Average duration of calls (±Std Dev.) for different categories of calls.

Call Types	WTMean±Std Dev	KOMean±Std Dev	P value
Complex	0.036±0.030	0.043±0.042	0.054
Upward	0.013±0.005	0.012±0.011	0.382
Downward	0.027±0.025	0.034±0.030	0.103
Chevron	0.015±0.001	0.020±0.005	0.073
Short	0.006±0.001	0.007±0.001	0.416
Composite	0.027±0.007	0.034±0.024	0.570
Flat	0.026±0.001	0.025±0.002	0.576

There was no effect of genotype on duration of calls for any of those call subtypes.

### Body weight

**Figure 4:**
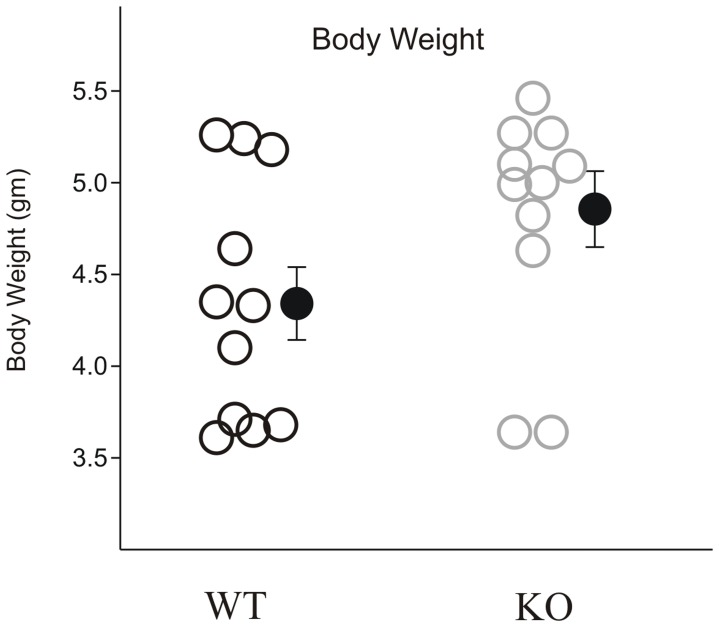
Body weight comparison between WT and in *Fmr1*-KO mice: Body weights of pups were measured on post natal day eight. Black circles: WT littermates; Gray circles: *Fmr1*-KO mice. Error bar represents standard error of the mean (SE). The body weights of mouse pups were not significantly different between genotypes.

The body weights of mouse pups tested for isolation induced USV calls were not significantly different between genotypes (t (20) = −1.864; P = 0.076; Figure 4) but there was a trend for *Fmr1*-KO pups to be lighter than their wild type littermates. Body weight was not correlated with the number of USV emitted (r = 0.353, p = 0.287).

## Discussion

Here we report that loss of *Fmr1* expression in a mouse model of fragile X syndrome does not cause a change in the number of isolation induced USVs generated by 8 day pups. Instead, we found that pups of *Fmr1* KO mice showed more subtle, call type specific deficits: the carrier frequency of flat calls was higher, the frequency range of complex calls was wider and the percentage of downward calls was lower in *Fmr1*-KO mice compared to their WT littermates.

Pups of *Fmr1*-KO mice emitted a lower percentage of downward calls compared to their WT littermates, while the percentages of all other call types were similar in both genotypes. Scattoni et al [14] performed a similar study on the BTR T+tf/j (BTBR) mouse strain that displays deficits in social behavior and repetitive behaviors analogous to the first and third diagnostic criteria of autism. Scattoni and colleagues compared BTBR mice with multiple standard strains including C57BL/6J, FVB/NJ and 129X1 and found that 8 day old BTBR pups produced the highest number of calls compared to all other strains. In BTBR mice, 3% of all calls were downward calls compared to 14% downward calls in C57BL/6J mice, a strain with a high level of social interaction. Thus, a reduced number of downward calls in isolation induced pup USVs might be correlated with deficits in social behavior. However, more mouse models with different levels of social behavior need to be examined to determine whether this variable is useful as a phenotypic signature of social interest or interaction in mouse models of autism spectrum disorders.

There was no genotype difference in the duration of calls between *Fmr1*-KO and WT pups. There are reports of longer and shorter duration of calls produced by different mouse models of autism [14], [16]. Call duration is socially relevant as the mother's behavioral response requires minimal call duration. Smith et al [17] showed that mothers preferred a call with a 80 ms duration over a call with a 15 ms duration. Ehret et al [18] found that mothers responded to calls with durations higher than 30 ms, but not to shorter ones. Thus, based on their call durations, *Fmr1*-KO mice are not expected to be at a disadvantage in eliciting the mother's response through USVs.

The average carrier frequency of flat calls emitted by KO mice were higher compared to their WT littermates. It is unclear if this change has an effect on the response of the mother. Mouse pup calls incorporate some properties that suggest they could serve some of the same functions as the crying of human babies, especially their abilities to elicit parental retrieval behaviors [19]–[23]. Researchers have been able to differentiate 80 measures of human infant crying, but frequency or pitch turned out to be the most important variable in facilitating adult recognition of infant needs [24]. Subjects who were later diagnosed with autism emitted higher pitched cries than those subjects with typical or delayed development, and autistic children's cries elicited greater negative states in their listeners [25]. Whether the elevated pitch in cries of autistic infants and the higher carrier frequency of flat USV calls of *Fmr1*-KO mice are functionally related or caused by related changes in neuronal control mechanisms remains to be shown.

Calls emitted by *Fmr1*-KO mice were frequency modulated over wider frequency ranges than calls emitted by WT pups. Brudzynski et al [26] postulated that the degree of frequency modulation could be important for the efficacy of maternal search and retrieval behavior. According to Wohr et al. [9], it may be easier for the mother to detect and localize a highly frequency modulated call than a steady sound at a constant frequency. In humans, the SHANK gene family has been associated with autism [27], [27,28]. Wohr and colleagues found isolation induced USV calls emitted by pups of Shank1^−/−^ mutant mice to be less frequency modulated than the calls of their WT littermates. This is in contrast to our findings that pups of *Fmr1*-KO mice have increased frequency modulation compared to their WT littermates, a difference that remains to be explained. However, the findings indicate that autism related changes in the genetic makeup of the nervous system might affect the control mechanisms for USVs. Linking the specific USV deficits reported here and in other studies to specific neuronal or genetic mechanisms will require more in-depth studies. Currently, the analysis of USVs in mouse models of autism spectrum disorders is still in its infancy.

Body weight has been speculated to affect USVs, e.g. through differences in lung capacity [14]. Here we can exclude an effect of bodyweight on our results as there was no difference in body weight at the time of testing between *Fmr1* KO and WT mice.

The neuronal mechanisms controlling human speech articulation and mouse oromotor/vocalization behavior are poorly understood. Several lines of evidence suggest that the cerebellum may play an important role in oromotor and vocalization/articulation in both species. In mice, the cerebellum has been shown to be critically involved in generation of ultrasonic vocalizations [29]. In humans, speech articulation deficits (dysarthria) are common in patients with cerebellar disorders [30]. Cerebellar neuropathologies, which are consistently found in fragile X patients [31]–[33] , might be partially responsible for speech articulation deficits in FXS patients [34]–[37]. In previous studies, we found oromotor deficits in a *Ube3a* deficient mouse model of Angelman syndrome [38] and in *Fmr1* KO mice [39]. Sensory mapping studies and recordings in awake behaving rodents have shown that the orofacial area is strongly represented in the cerebellum of rats and mice [40]–[42]. Cerebellar deficits, manifest in the reduced volume of the medial and interposed nuclei of *Fmr1*-KO mice [43], are thus likely to contribute to the subtle USV deficits in mouse pups described here.
